# Interactive webtool for analyzing drug sensitivity and resistance associated with genetic signatures of cancer cell lines

**DOI:** 10.1007/s00432-022-04503-2

**Published:** 2022-12-06

**Authors:** Myriam Boeschen, Diana Le Duc, Mathias Stiller, Maximilian von Laffert, Torsten Schöneberg, Susanne Horn

**Affiliations:** 1 Rudolf Schönheimer Institute of Biochemistry, Medical Faculty, University of Leipzig, Johannisallee 30, 04103 Leipzig, Germany; 2 Institute of Human Genetics, University of Leipzig Medical Center, Leipzig, Germany; 3 Institute of Pathology, University Hospital Leipzig, Leipzig, Germany; 4 Department of Dermatology, University Hospital Essen, University Duisburg-Essen, and German Cancer Consortium (DKTK) Partner Site Essen/Düsseldorf, 45122 Essen, Germany

**Keywords:** Association test, Candidate compound, Data repository, Discovery approach

## Abstract

**Purpose:**

A wide therapeutic repertoire has become available to oncologists including radio- and chemotherapy, small molecules and monoclonal antibodies. However, drug efficacy can be limited by genetic heterogeneity. Here, we designed a webtool that facilitates the data analysis of the in vitro drug sensitivity data on 265 approved compounds from the GDSC database in association with a plethora of genetic changes documented for 1001 cell lines in the CCLE data.

**Methods:**

The webtool computes odds ratios of drug resistance for a queried set of genetic alterations. It provides results on the efficacy of single compounds or groups of compounds assigned to cellular signaling pathways. Webtool availability: https://tools.hornlab.org/GDSC/.

**Results:**

We first replicated established associations of genetic driver mutations in *BRAF*, *RAS* genes and *EGFR* with drug response. We then tested the ‘BRCAness’ hypothesis and did not find increased sensitivity to the assayed PARP inhibitors. Analyzing specific *PIK3CA* mutations related to cancer and mendelian overgrowth, we found support for the described sensitivity of H1047 mutants to GSK690693 targeting the AKT pathway. Testing a co-mutated gene pair, *GATA3* activation abolished *PTEN*-related sensitivity to PI3K/mTOR inhibition. Finally, the pharmacogenomic modifier *ABCB1* was associated with olaparib resistance.

**Conclusions:**

This tool could identify potential drug candidates in the presence of custom sets of genetic changes and moreover, improve the understanding of signaling pathways. The underlying computer code can be adapted to larger drug response datasets to help structure and accommodate the increasingly large biomedical knowledge base.

**Supplementary Information:**

The online version contains supplementary material available at 10.1007/s00432-022-04503-2.

## Introduction

**Fig. 1:**
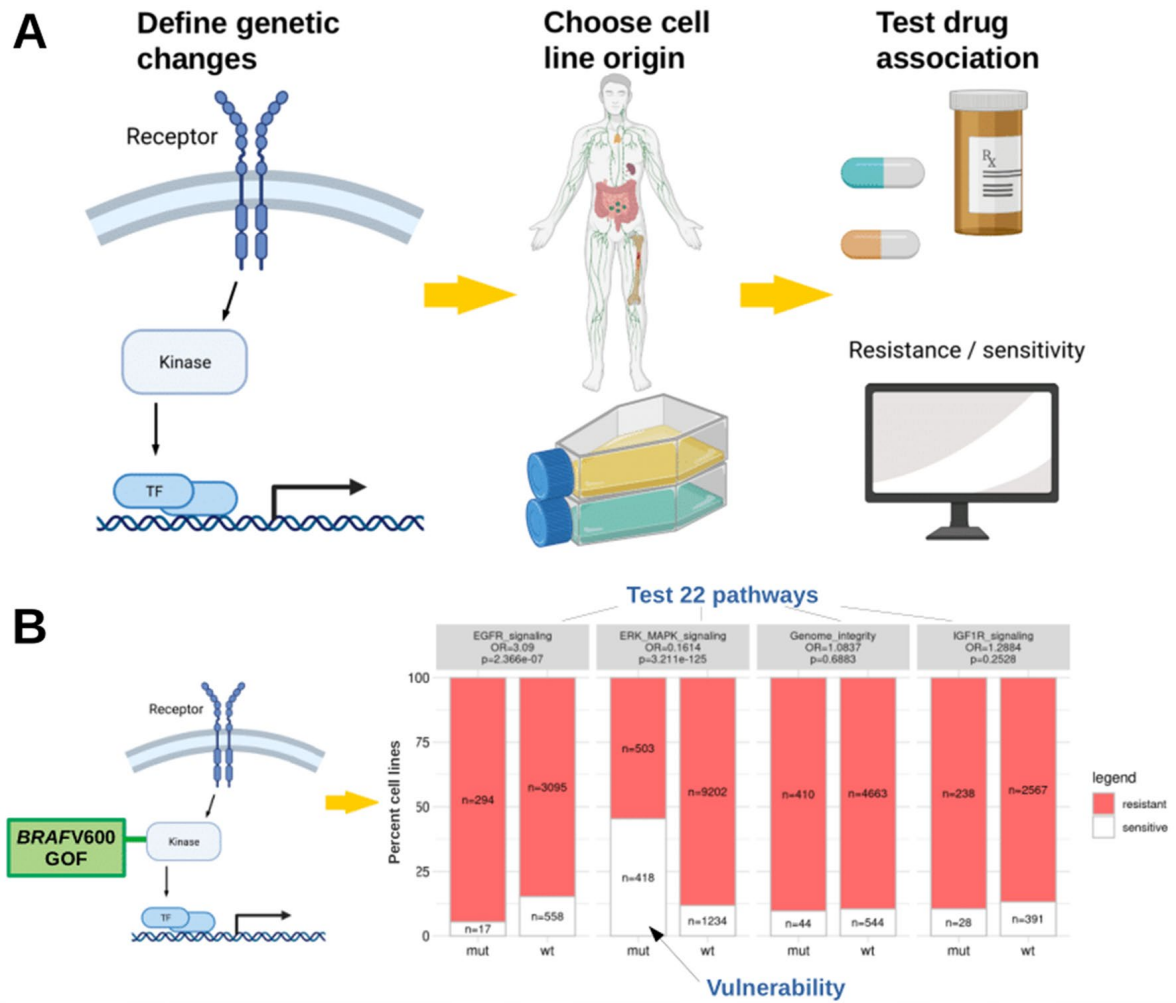
Workflow of the webtool and verification of the *BRAF*–MAPK pathway association. **A** Users define custom genetic changes and test across cell lines from various cancer entities. Categorization in ‘resistant’ or ‘sensitive’ from Iorio et al*.* (Iorio et al. 2016): cell viability measured using the CellTiter-Glo^®^ Assay, IC50 values subsequently estimated by a non-linear mixed effects model, binarization threshold for each drug. **B** Principle of testing the gain of function (GOF) *BRAF* V600-mutated cell lines revealing moderate resistance to EGFR and strong sensitivity to ERK/MAPK pathway compounds. Odds ratios (OR) for resistance in the altered cell lines (mut) above each bar plot (Fisher’s tests). Numbers of cell lines pooled in each pathway category differ by available data indicated in the bars. Using Bonferroni correction for testing 22 pathways, *p*-values < 0.0023 are considered significant

Genetic diversity across cancers modifies the tumor’s drug responsiveness and impedes the use of widely applicable drugs without patient stratification (Lin and Sheltzer 2020). The *BRAF* V600-specific inhibition of tumor growth across various tumor entities is a prime example of personalized cancer treatment (Subbiah et al. 2020) and depends on the availability of diagnostic biomarkers with predictive power for drug response (Chin et al. 2011). To identify and establish those powerful decisive markers, it is crucial to associate specific genetic changes with drug response data. Complex datasets have been generated for cancer cell lines to provide information on genetic changes and drug response. The Genomics of Drug Sensitivity in Cancer Project (GDSC) and The Cancer Cell Line Encyclopedia (CCLE) represent two of such databases (Iorio et al. 2016; Ghandi et al. 2019). While GDSC provides drug response data including bioinformatically estimated concentration–response curves, IC_50_ values as well as tested genomic associations, the CCLE dataset holds information on altered gene copy number, point mutations, mRNA levels, and gene fusions. Although public webtools were developed for ready access and analysis of the above datasets (Cerami et al. 2012; Yang et al. 2013; Piñeiro-Yáñez et al. 2018; Basu et al. 2013; Najgebauer et al. 2020), these tools often lack the possibility to analyze a custom set of mutations or a combination of co-occurring gene alterations. Here, we designed a straightforward publicly accessible tool that combines the GDSC drug response data and genetic data collected for the CCLE samples. It enables researchers to query individual combinations of genetic alterations to assess drug sensitivity for a larger variety of cell lines (Fig. 1A). Our approach allows any combination of genetic changes in the query genes to address cellular pathways. Since different genetic changes of the same gene can mediate various functional effects, we offer custom queries for hypothesis testing and discovery of drugs for potential repurposing.

## Materials and methods

### Implementation

**Fig. 2:**
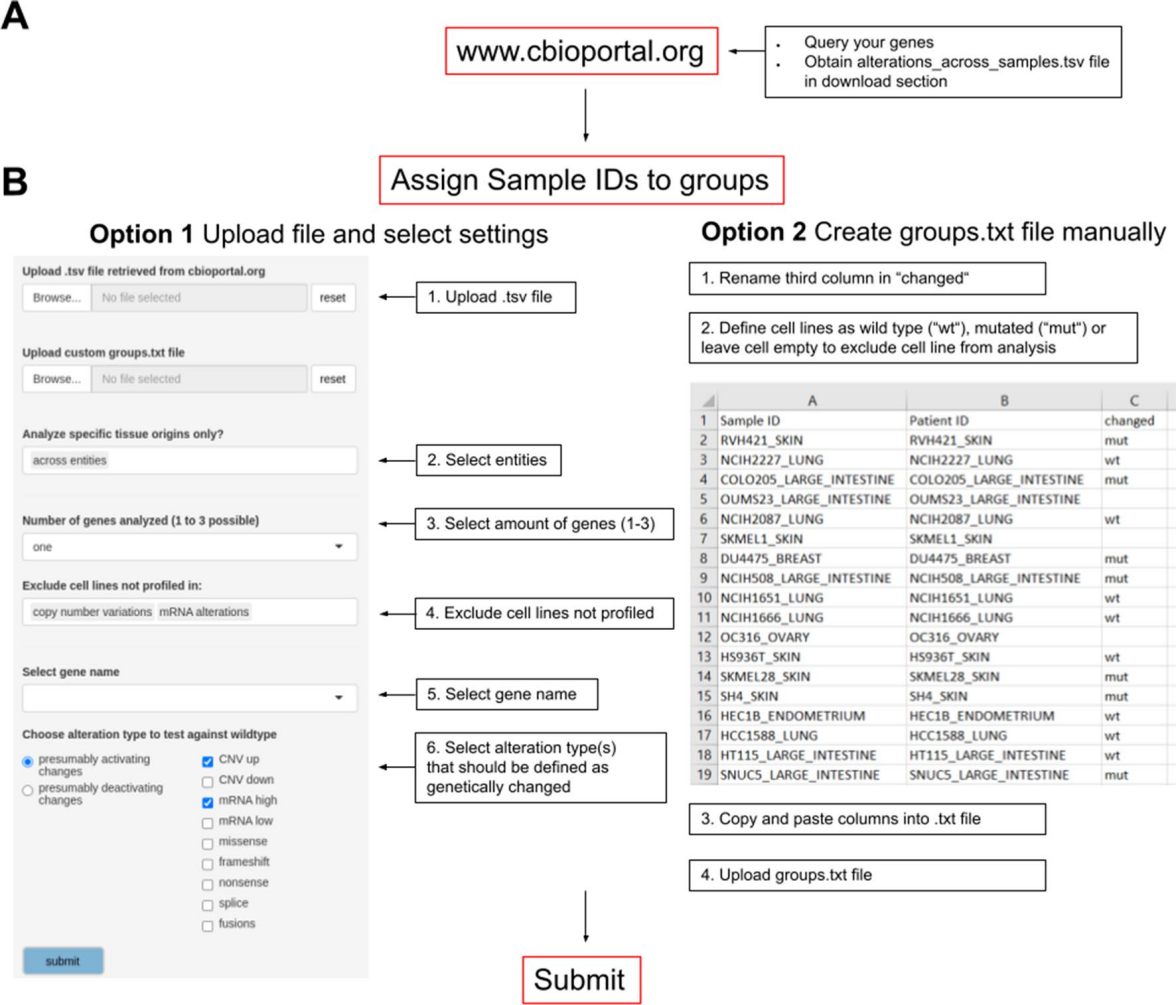
Step-by-step user workflow. **A** Obtain genetic alterations of query genes in the ‘alterations_across_samples.tsv’ file from www.cBioPortal.org as described at https://tools.hornlab.org/GDSC/Manual.pdf and in Supplementary Fig. 1–2. **B** Based on this file, cell lines are classified in ‘genetically changed’ and ‘wild type’ as reference. Option 1: tumor entities (tissue origins) of interest can be selected. Up to three genes can be chosen for analysis. The webtool provides automated assignment of cell lines into groups, by default ‘presumably activating’: amplifications (CNV up), high mRNA as defined by cBioportal. Default: ‘presumably deactivating’: homozygous deletions (CNV down), low mRNA, nonsense- and frameshift mutations. Users can additionally select missense mutations, splice variants and gene fusions. For two or three genes, cell lines can be defined as ‘mutated’ if at least one of the genes is changed or optionally, if all genes are changed (co-mutation). Option 2: custom grouping based on a tab-delimited text file and used in the upload option of the webtool. It should contain 3 columns named ‘Sample.ID’, ‘Patient.ID’, and ‘changed’. The first two columns are retrieved from the ‘alterations_across_sample.tsv’ file and the third column, added by the user, defines the cell lines as ‘mut’ for mutated/genetically changed and ‘wt’ for wild type (empty cells to exclude cell lines)

The webtool was implemented using R shiny in R version 4.0.5. To avoid duplications of 15 compounds screened twice, the screenings with higher data availability were used. Drugs were assigned to one of 22 target pathways as previously described (Iorio et al. 2016). Further information on compounds can be retrieved in the GDSC database at https://www.cancerrxgene.org/ and the compounds of the GDSC1 dataset are included in our webtool. Here, we analyzed genes across all available cell lines as the majority of cell lines were profiled for the queried alterations. The ‘BRCAness’ analysis was additionally performed on breast cancer cell lines only. Users can specify which genetic changes and cell lines shall be included in the analysis, for every gene the type of genetic changes can be selected independently. Furthermore, cell lines with missing data, which were not profiled for a specific type of alteration, can be excluded to avoid ascertainment biases in the analyses, e.g., of gene fusions which have not been profiled for the majority of cell lines (Fig. 2). Plots and data tables are provided for download.

### Statistical analysis

From the contingency tables of genetically changed and wild-type groups, odds ratios (OR) for resistance and Fisher’s exact tests (two-sided) were computed, within each pathway as well as for every single compound. Bonferroni-corrected *p* value thresholds are < 0.0023 testing 22 pathways and *p* < 0.0002 testing 250 drugs. For counts of zero, it was not possible to calculate OR and ratios of resistant and sensitive cell lines are shown in percent above the bar plot.

## Results and discussion

As a proof of principle, we tested established gene–drug associations across tumor entities. Specific gene mutations are routinely used as biomarkers to guide targeted therapies, such as *BRAF* V600 mutations for the targeted MAP kinase inhibition (MAPKi) therapy of cancer patients (Subbiah et al. 2020). We replicated this established association with strong statistical support in our webtool. *BRAF* V600-positive cell lines were sensitive to compounds targeting the ERK–MAPK signaling pathway (OR 0.16, *p* = 3.2e−125, Fig. 1B) such as PLX4720 (OR 0.03, *p* = 6.2e−38) and dabrafenib (OR 0.02, *p* = 3.87e−36, Supplementary Fig. 3). Of note, *BRAF* V600-mutated cell lines were resistant to compounds targeting the PI3K/mTOR pathway (OR 1.72, *p* = 1.12e−7), EGFR pathway (OR 3.10, *p* = 2.37e−7), and the RTK pathway (OR 1.82, *p* = 1.47e−9, Supplementary Fig. 4A).

**Fig. 3:**
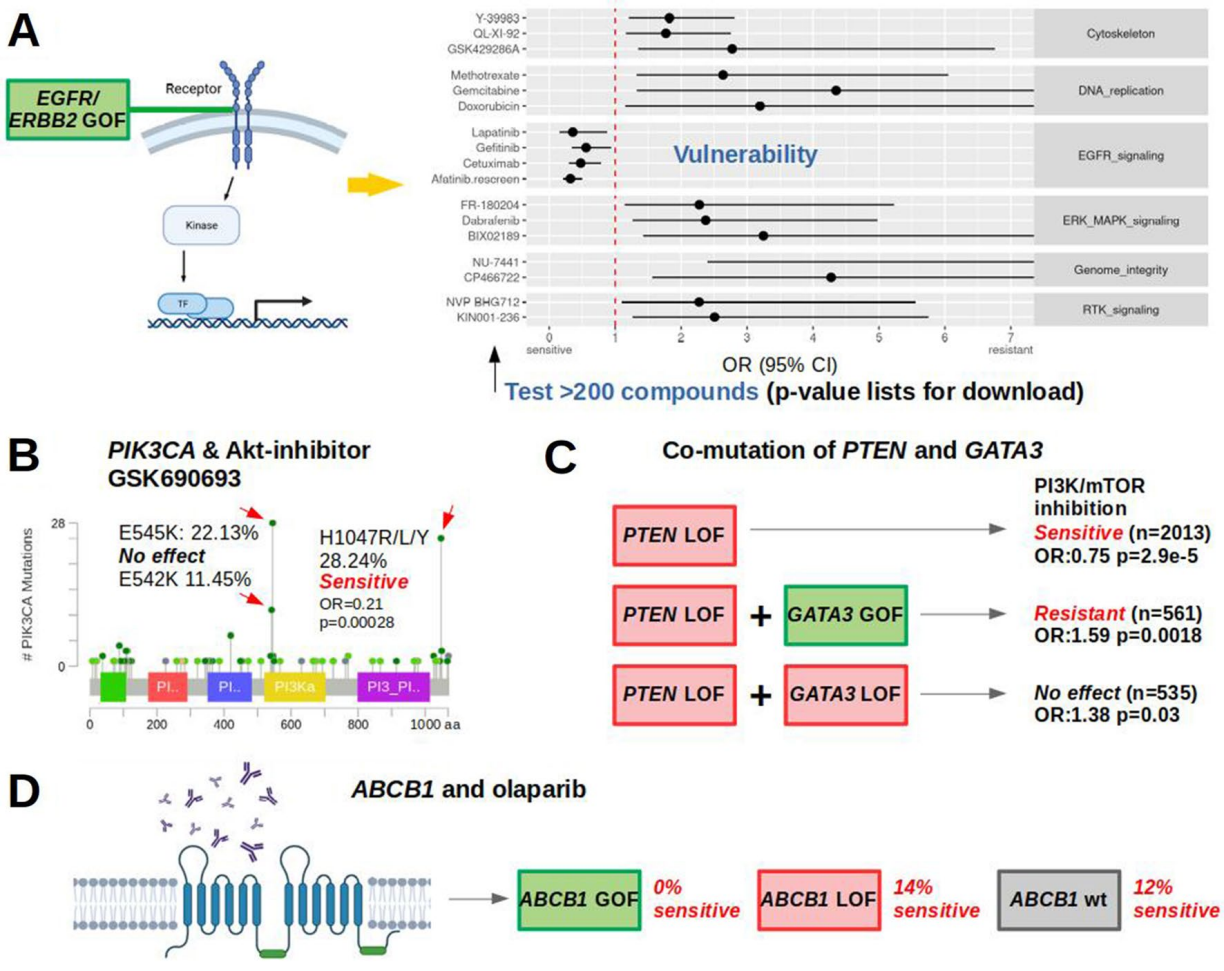
Verification of EGFR pathway association and new drug dependencies. **A** Compounds that show activity in *EGFR/ERBB2*-activated cell lines almost exclusively belong to the EGFR pathway. Forest plot with OR > 1 shows resistance of altered cell lines to drugs, OR < 1 indicates sensitivity. Compounds shown if *p* < 0.05 from Fisher’s exact tests for candidate approaches. For discovery testing 250 compounds, Bonferroni-corrected *p* < 0.0002 are considered significant. **B** Within frequent *PIK3CA* point mutations only H1047 mutant cell lines were sensitive to AKT-inhibitor GSK690693 (Supplementary Table 3). Frequencies and image for CCLE 2019 from www.cbioportal.org. Colored regions: protein domains. Light green: missense variants of unknown significance, dark green: driver missense mutations. **C** *GATA3* activation and abolished *PTEN*-related sensitivity to PI3K/mTOR inhibition. **D** Model of olaparib exclusion with activated *ABCB1*. GOF/LOF: gain/loss of function

Since RAS proteins activate the MAPK-signaling pathway (Bonni et al. 1999), we expected cancer cell lines carrying one of the recurrent point mutations in *NRAS*, *HRAS* or *KRAS* to be sensitive to compounds targeting the ERK–MAPK signaling pathway as well. This alleged association was confirmed across entities with great statistical support (OR 0.49, *p* = 3.71e−33, Supplementary Fig. 4B). As the role of *EGFR* and *ERBB2* overexpression and amplification in cancer is well described (Olayioye et al. 2000), we expected to replicate a sensitivity to compounds inhibiting the EGFR-signaling pathway in *EGFR-* or *ERBB2-*altered cell lines. Cell lines with potentially activating changes including gene amplifications as well as high mRNA levels in either *EGFR* or *ERBB2*, or both genes were associated with sensitivity to EGFR pathway compounds (OR 0.51, *p* = 3.05e−8, Supplementary Fig. 5). Compounds that showed sensitivity in *EGFR/ERBB2-*altered cell lines almost exclusively target the EGFR pathway (Fig. 3A). Furthermore, as PTEN loss results in hyperactive PI3K signaling (Carracedo and Pandolfi 2008) we tested cell lines with *PTEN* deactivating changes (homozygous deletions, splice variants, frameshift and nonsense mutations) and confirmed those to be sensitive to compounds inhibiting the PI3K/mTOR pathway (OR 0.75, *p* = 2.94e−5, Supplementary Fig. 6A, B). Hence, we replicated several established associations of genetic changes and drug sensitivity across cancer entities, indicating the reliability of the presented webtool to detect true strong associations.

Next, we tested proposed gene–drug associations, at first the ‘BRCAness’ hypothesis (Lord and Ashworth 2016), comparing cancer cell lines with deactivating changes (homozygous deletions, splice variants, frame shift, and nonsense mutations) in at least one of the following ‘BRCAness’ genes: *BRCA1*, *BRCA2*, *ATM*, *ATR*, *RAD51C*, and *RAD51D* with wild types across entities. The sensitizing effect of *BRCA* deactivation for poly (ADP-ribose) polymerase inhibition (PARPi) therapy has been shown multiple times across cancer entities (Fong et al. 2009). However, we found no evidence for an increased sensitivity to the assayed PARP inhibitors neither across entities nor in breast cancer cell lines (Supplementary Table 2). These results indicate that ‘BRCAness’ may not be an obligate prerequisite for benefit from PARPi in line with recent studies (Shen et al. 2019). As a second test of a previously reported association we tested PI3K/AKT/mTOR inhibitors and *PIK3CA* mutations. Somatic mutations have been reported to occur in cancer as well as pathogenic germline variants in *PIK3CA*-related overgrowth. We tested a set of 21 drugs from the group of PI3K/AKT/mTOR inhibitors and observed support for the described sensitivity of the H1047 mutant cell lines (Janku et al. 2013) for at least one inhibitor in the pathway, the AKT-inhibitor GSK690693 (OR 0.21, *p* = 0.00028 at alpha = 0.002, Fig. 3B, Supplementary Table 3). Furthermore, *PIK3CA* E545K or E542K mutated cancer cell lines had been described to be less sensitive (Janku et al. 2013) and indeed were not associated with an apparent response to GSK690693 (OR 0.81, *p* = 0.69). Beyond cancer, the mutational spectra of *PIK3CA*-related cancer and *PIK3CA*-related overgrowth syndromes (PROS) overlap (Venot et al. 2018). Patients with PROS showed improvements in disease symptoms following treatment with the PI3K inhibitor BYL719 and we, thus, tested the most frequent PROS *PIK3CA* mutations H1047, E542 and C420. Although BYL719 is not included in our drug dataset, cell lines harboring one of the 3 mutations also showed sensitivity to the Akt inhibitor GSK690693 (OR 0.28, *p* = 0.00019). Hence, we support the notion that blocking Akt is efficient in the presence of one of the three most frequent *PIK3CA*-overgrowth mutations.

To test drug resistance of co-mutations in a large cancer entity we chose the significantly co-mutated gene pair *PTEN* and *GATA3* (co-mutation *p* < 0.001, Supplementary Fig. 6C–E). *GATA3* expression was described to delay tumor progression and reduce Akt activation in *PTEN*-deficient mouse prostate cancer (Nguyen et al. 2013) and resulted in differential drug sensitivity in breast cancer (Mair et al. 2016). While here, *PTEN* deactivation was again associated with the previously observed sensitivity to compounds targeting the PI3K/MTOR pathway, a concomitant *GATA3* activation (amplification, mRNA high) conferred resistance (OR 1.59, *p* = 0.0018, Fig. 3C**,** Supplementary Table 4). A concomitant *GATA3* deactivation (homozygous deletions, splice variants, frameshift and nonsense mutations) did not yield a significant result (OR 1.38, *p* = 0.029, significance level testing 22 pathways: 0.002) although an opposite effect could be expected from *GATA3* deactivation leading to either no changes in PI3K/mTOR inhibition retaining sensitivity or even enhancing sensitivity. These results may prompt more experiments to test if *GATA3* is indeed functionally involved in the PTEN-axis of PI3K/mTOR inhibitor sensitivity.

Next, we tested putatively activating genetic changes of the pharmacogenomic modifier *ABCB1* vs. the wild type and found resistance to compounds from various pathways (Supplementary Table 5). Olaparib efflux has been reported for advanced prostate cancer over-expressing *ABCB1* (Lombard et al. 2019) and also here all of the *ABCB1*-activated cell lines were resistant to olaparib (*n* = 76, *p* = 0.00015, Fig. 3D), while 12.4% of wild-type cell lines were sensitive. A deactivation of *ABCB1* in 21 cell lines had no significant association with olaparib efficacy (OR 0.82, *p* = 0.7373).

## Conclusion

In conclusion, we present a powerful webtool to analyze associations of genetic alterations and drug response from the CCLE and GDSC datasets and enable fast hypothesis testing and discovery approaches across and within cancer entities. The swift analysis of molecular tumor heterogeneity may help to identify compounds for drug repurposing and prioritization and to understand settings of co-mutation. More broadly, beyond cancer it could be applied to investigate mechanisms of cell signaling pathways. As the public data sets are currently growing fast, larger sample sizes will improve statistical power, McInnes et al*.* recently released a pharmacogenetic analysis including 487,409 participants (McInnes, Lavertu et al. 2021). The open source of our webtool is technically applicable to these larger datasets with binary and continuous measures for drug response. Hence, it will be possible to further expand it as a functional tool to bridge from big drug sensitivity screens to bench-side researchers.

## Supplementary Information

Below is the link to the electronic supplementary material.

Supplementary file1 (PDF 2883 KB)

Supplementary file2 (DOCX 13 KB)

## Data Availability

Generated code, group definitions of cell lines and resulting tables are available at https://github.com/MyriamBoeschen/Drug_Response_Tool, data sources in Supplementary Table 1.
