# Supplementary material for: Interactive webtool for analyzing drug sensitivity and resistance associated with genetic signatures of cancer cell lines
Source: J Cancer Res Clin Oncol. 2022 Dec 6;149(9):5539–45. doi: 10.1007/s00432-022-04503-2 (PMC10356876; doi:10.1007/s00432-022-04503-2)
Supplement: Supplementary file 1 — Supplementary file1 (PDF 2883 KB) [file 432_2022_4503_MOESM1_ESM.pdf]

How to retrieve the alterations_across_samples.tsv file for your analysis

→ go to https://www.cbioportal.org/

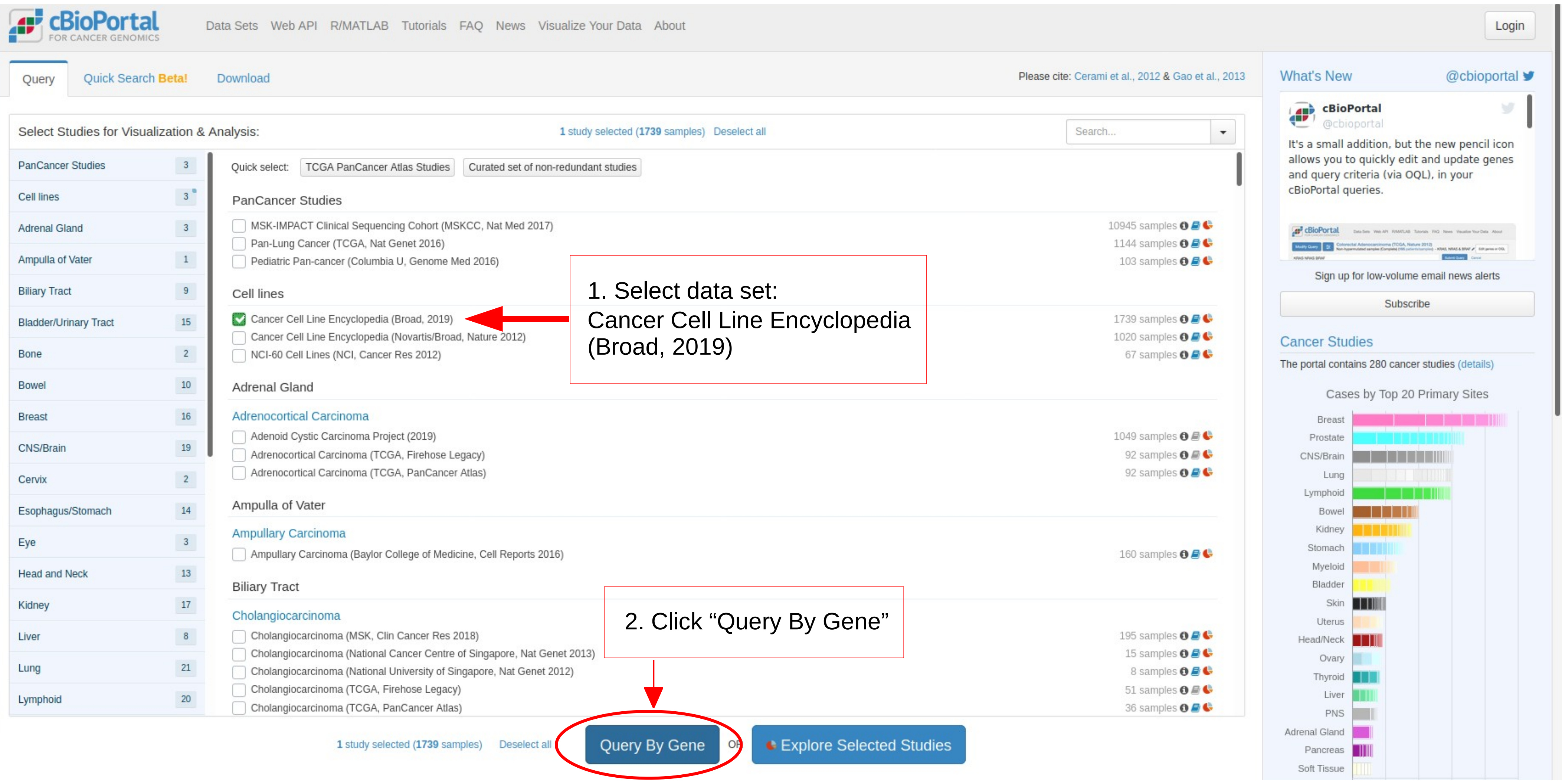

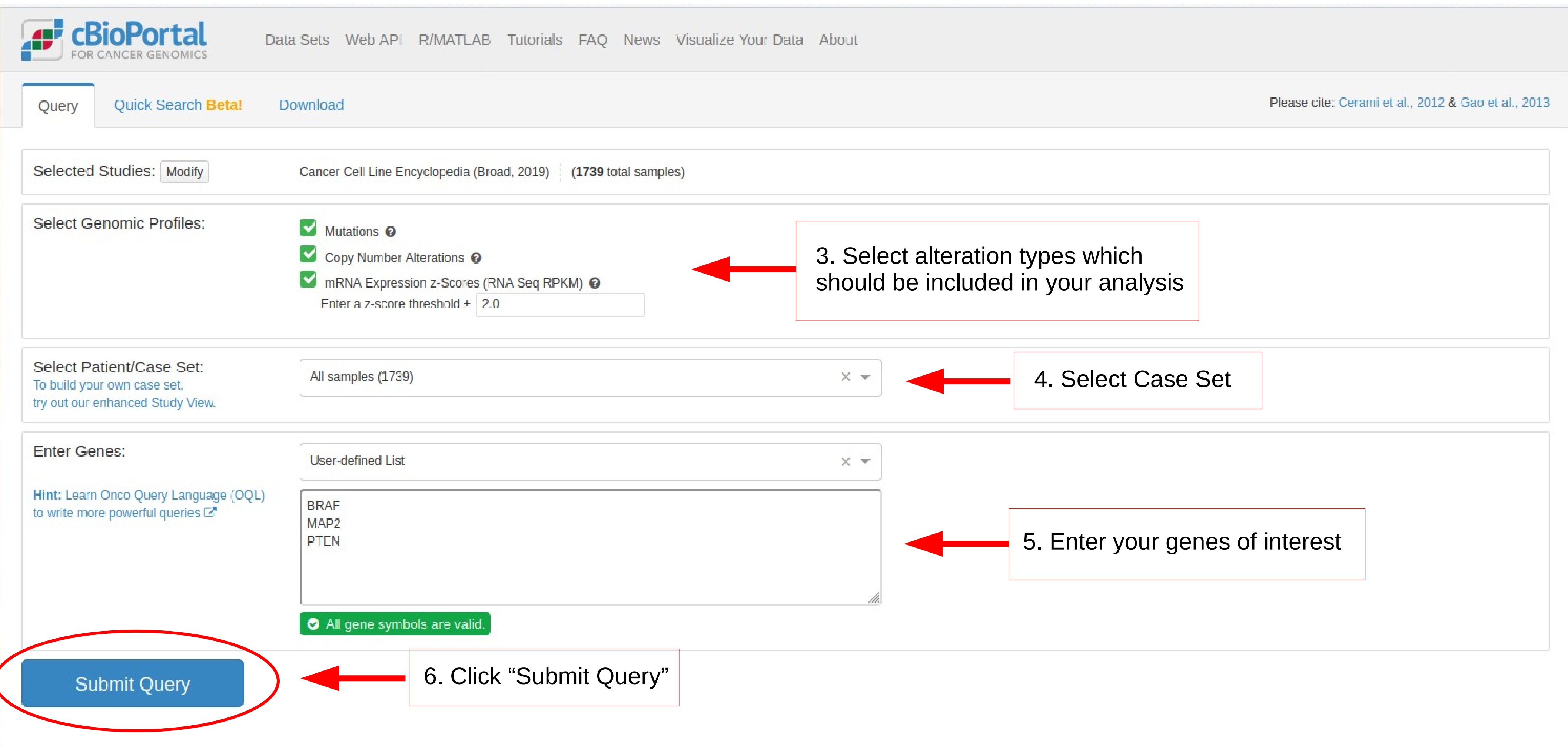

Supplementary Figure 1. Step-by-spec guide how to retrieve the ‘alterations_across_samples.tsv’ file from https://www.cbioportal.org/ part 1. After selecting the Cancer Cell Line Encyclopedia (Broad, 2019), click ‘Query by Gene’. On the following page genomic alteration types can be chosen and and genes of interest entered.

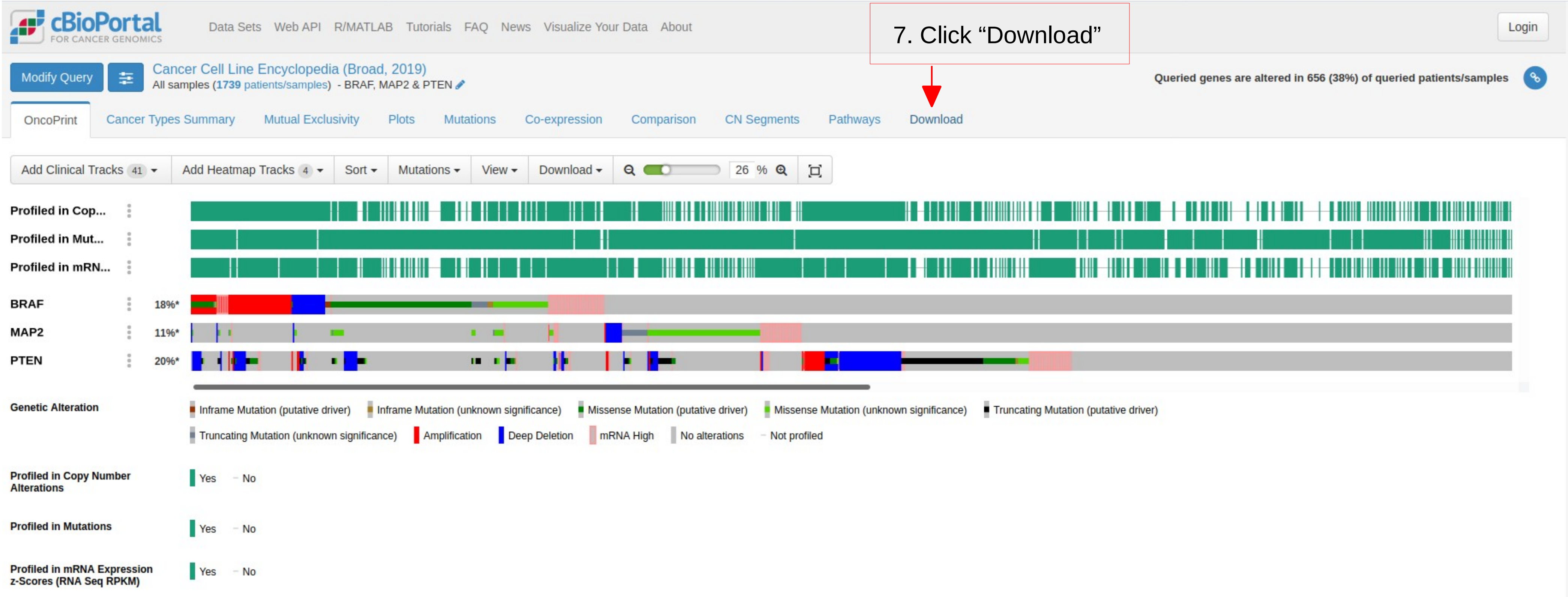

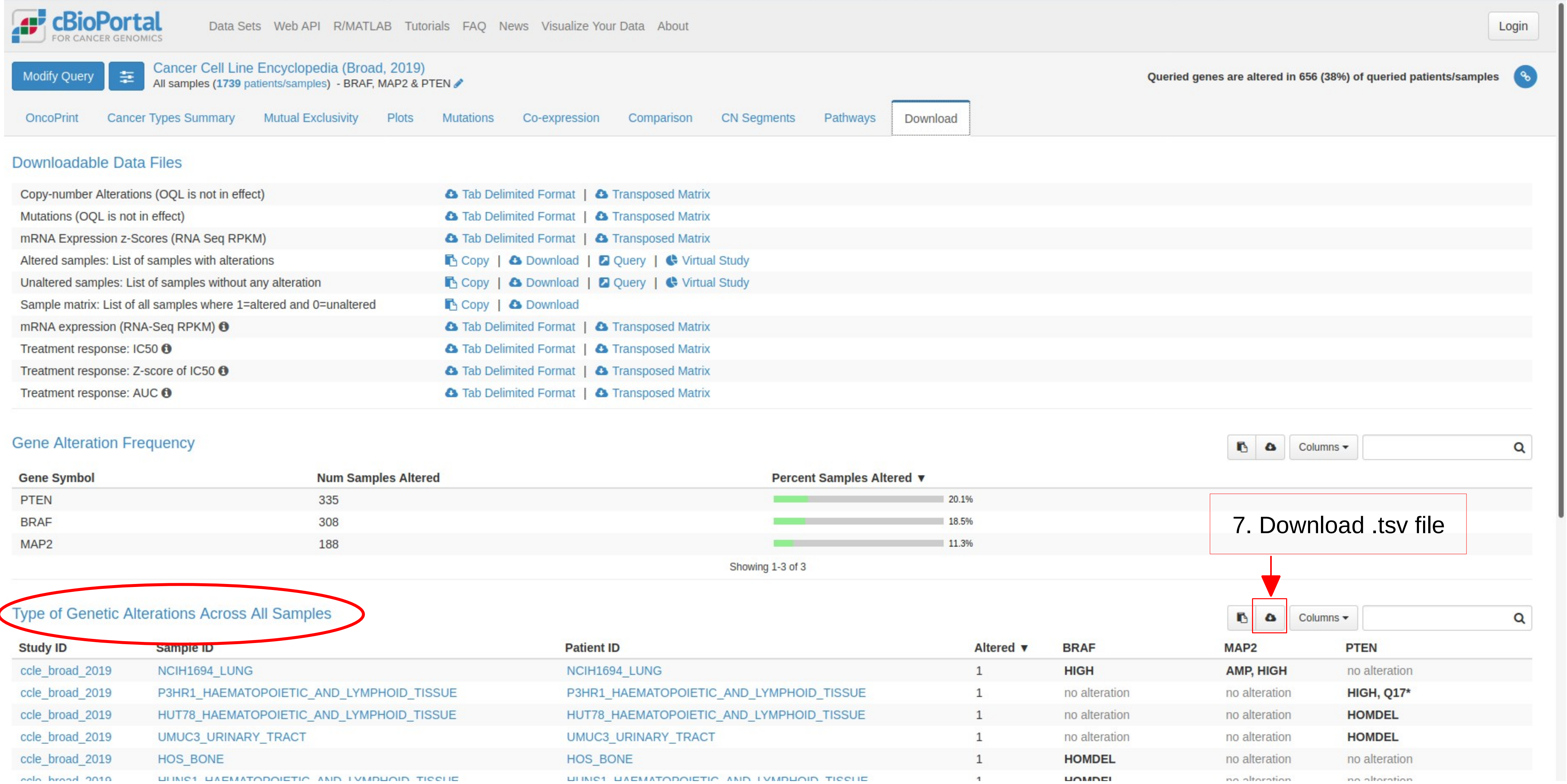

Supplementary Figure 2. Step-by-spec guide how to retrieve the ‘alterations_across_samples.tsv’ file from cBioPortal part 2. Under the last tab ‘Download’, the section ‘Type of Genomic Alterations Across All Samples’ can be found and the ‘alterations_across_samples.tsv’ file can be downloaded with the cloud symbol on the right site.

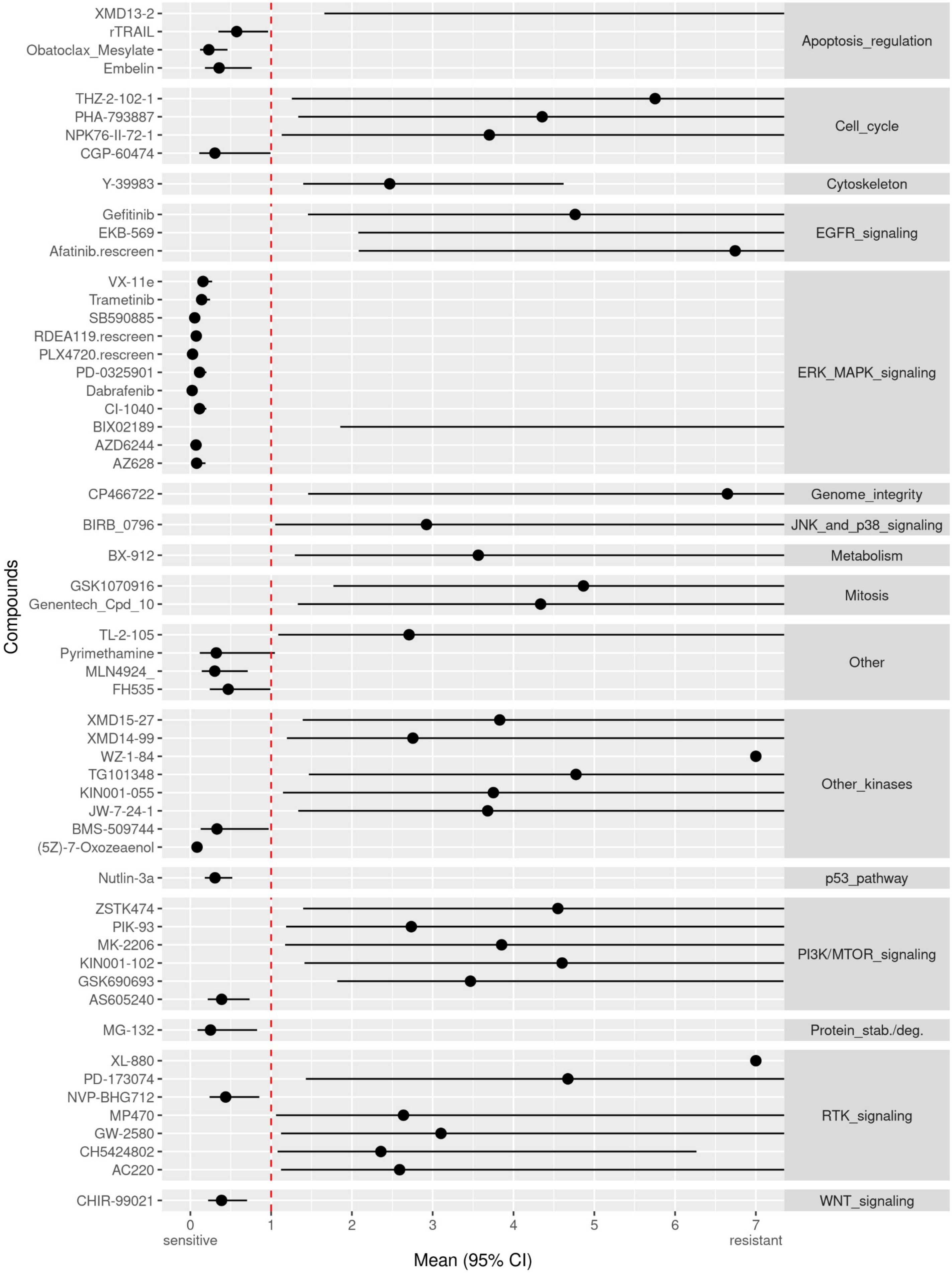

Supplementary Figure 3. Associations of drug response to single compounds and BRAFV600 mutations. Forest plot with OR and confidence intervals given for compounds with $p<0.05$ (fisher's exact tests). Comparisons with groups of the size zero with infinite confidence intervals are depicted as single points at OR=0 (sensitive) or OR=7 (resistant). Analysis performed across entities.

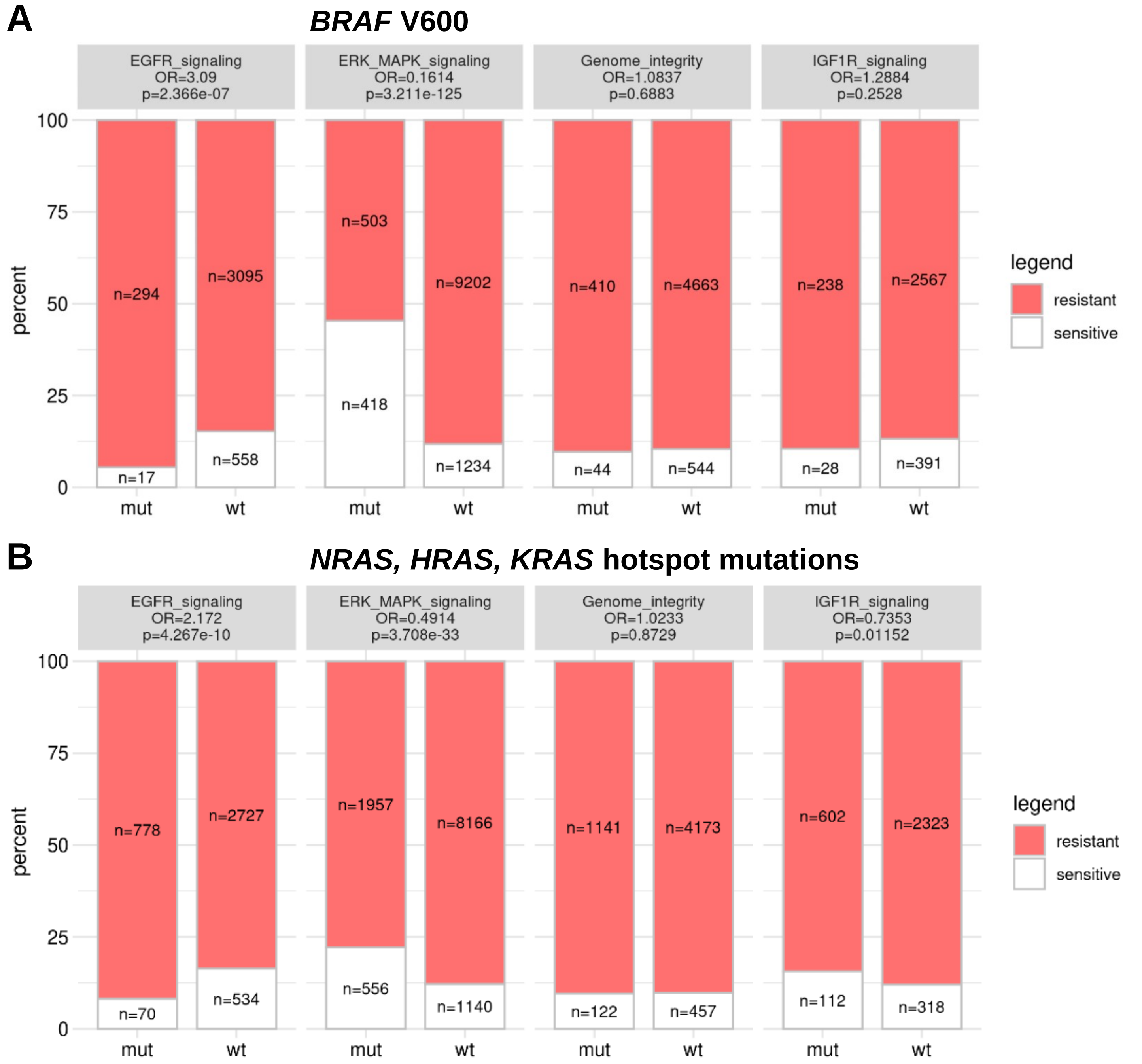

Supplementary Figure 4. Cell lines with activating changes in *BRAF* V600 (A) and in *RAS* genes (B) show sensitivity to ERK/MAPK inhibition. For each pathway (grey boxes) two barplots show numbers of cell lines pooled in the changed/mutated group (mut) and in the wild type (wt). Percent of resistant (red) and sensitive (white) cell lines indicated on the y-scale. Odds ratios (OR) for resistance in the altered cell lines above each bar plot: OR >1: resistance of altered cell lines to drugs in this pathway, OR<1: sensitivity.

A

drug_group	OR	fisher_p	chi_p	lower	upper
Other_kinases	1.7062	6.284E-18	1.637E-16	1.5033	1.944
Other	1.4415	4.928E-10	1.518E-09	1.2813	1.6268
PI3K/MTOR_signaling	1.547	2.377E-08	6.958E-08	1.3219	1.8208
EGFR_signaling	0.5125	3.045E-08	5.1E-09	0.4093	0.6453
Chromatin_other	2.4925	7.835E-08	4.298E-07	1.753	3.6651
Cell_cycle	1.8093	3.169E-07	1.087E-06	1.4297	2.3241
DNA_replication	1.8182	1.372E-06	3.926E-06	1.415	2.3747
ERK_MAPK_signaling	1.4639	4.555E-06	7.653E-06	1.2407	1.7372
Cytoskeleton	1.6085	9.924E-06	1.556E-05	1.2993	2.0104
Chromatin_histone_acetylation	1.5991	1.753E-05	3.044E-05	1.286	2.011
p53_pathway	2.3501	2.495E-05	5.751E-05	1.5588	3.7104
Metabolism	2.0327	2.846E-05	7.154E-05	1.4407	2.9643
Genome_integrity	1.8228	3.651E-05	7.339E-05	1.361	2.4962
RTK_signaling	1.3427	5.285E-05	7.49E-05	1.1621	1.5585
Apoptosis_regulation	1.4639	0.001347	0.00156	1.16	1.8705
Mitosis	1.4984	0.001367	0.00174	1.1684	1.9519
JNK_and_p38_signaling	1.5021	0.006234	0.006583	1.1263	2.0447
IGF1R_signaling	1.3581	0.06166	0.05772	0.9956	1.8926
Protein_stab./deg.	1.2857	0.1425	0.1287	0.9358	1.8121
Chromatin_histone_methylation	1.4579	0.2131	0.1678	0.8656	2.6399
ABL_signaling	1.2925	0.4237	0.3568	0.7552	2.3686
WNT_signaling	1.0888	0.6632	0.6223	0.7747	1.566

B

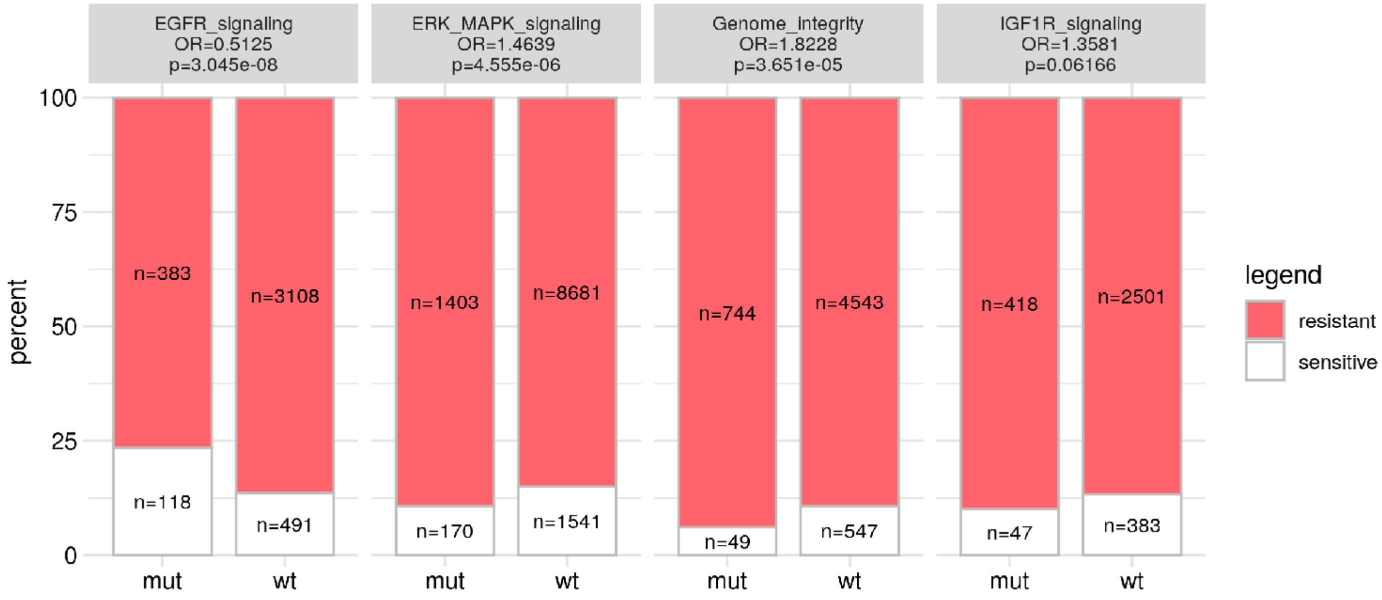

Supplementary Figure 5. Cell lines with activating changes in *EGFR* or *ERBB2* show enhanced sensitivity to compounds targeting EGFR signaling. Analysis of activating changes (amplifications, mRNA high z-scores relative to diploid samples with z-score ± 2) across entities. **(A)** Sensitivity to a single pathway is observed with significance (highlighted in yellow) while altered cell lines appear resistant to most drugs from other pathways. **(B)** Odds ratios (OR) for resistance in the altered cell lines shown above each barplot (OR>1 indicates resistance, OR<1 sensitivity.

A

drug_group	OR	fisher_p	chi_p	lower	upper
PI3K/MTOR_signaling	0.7473	3.26E-05	1.87E-05	0.6547	0.8554
IGF1R_signaling	1.8572	0.00048	0.00074	1.3048	2.7279
EGFR_signaling	1.569	0.00197	0.00225	1.1805	2.1232
Other	0.8624	0.00727	0.00646	0.7761	0.9602
RTK_signaling	0.8696	0.04557	0.04246	0.7611	0.9966
DNA_replication	1.2712	0.04825	0.04635	1.0075	1.6239
Cell_cycle	0.8388	0.08006	0.07331	0.6946	1.0197
Chromatin_histone_methylation	0.6564	0.09115	0.06961	0.4229	1.0533
Cytoskeleton	0.8482	0.102	0.0955	0.701	1.0317
Other_kinases	1.0789	0.1992	0.1906	0.9636	1.211
ERK_MAPK_signaling	1.1074	0.2082	0.2044	0.9471	1.3007
Apoptosis_regulation	0.896	0.331	0.3273	0.7236	1.1189
ABL_signaling	1.3101	0.4219	0.3347	0.7634	2.4057
Chromatin_histone_acetylation	1.0859	0.4422	0.4264	0.8873	1.34
Protein_stab./deg.	0.9001	0.481	0.4976	0.6736	1.2242
Chromatin_other	0.9378	0.6606	0.6645	0.7113	1.2532
p53_pathway	1.0698	0.7331	0.6865	0.7677	1.5225
JNK_and_p38_signaling	1.0241	0.891	0.8524	0.7862	1.3536
WNT_signaling	1.0386	0.9282	0.8226	0.7341	1.5039
Metabolism	1.0171	0.9395	0.8995	0.7599	1.3865
Genome_integrity	1.019	0.9481	0.8754	0.793	1.3265
Mitosis	0.9901	0.9538	0.9402	0.7931	1.2487

B

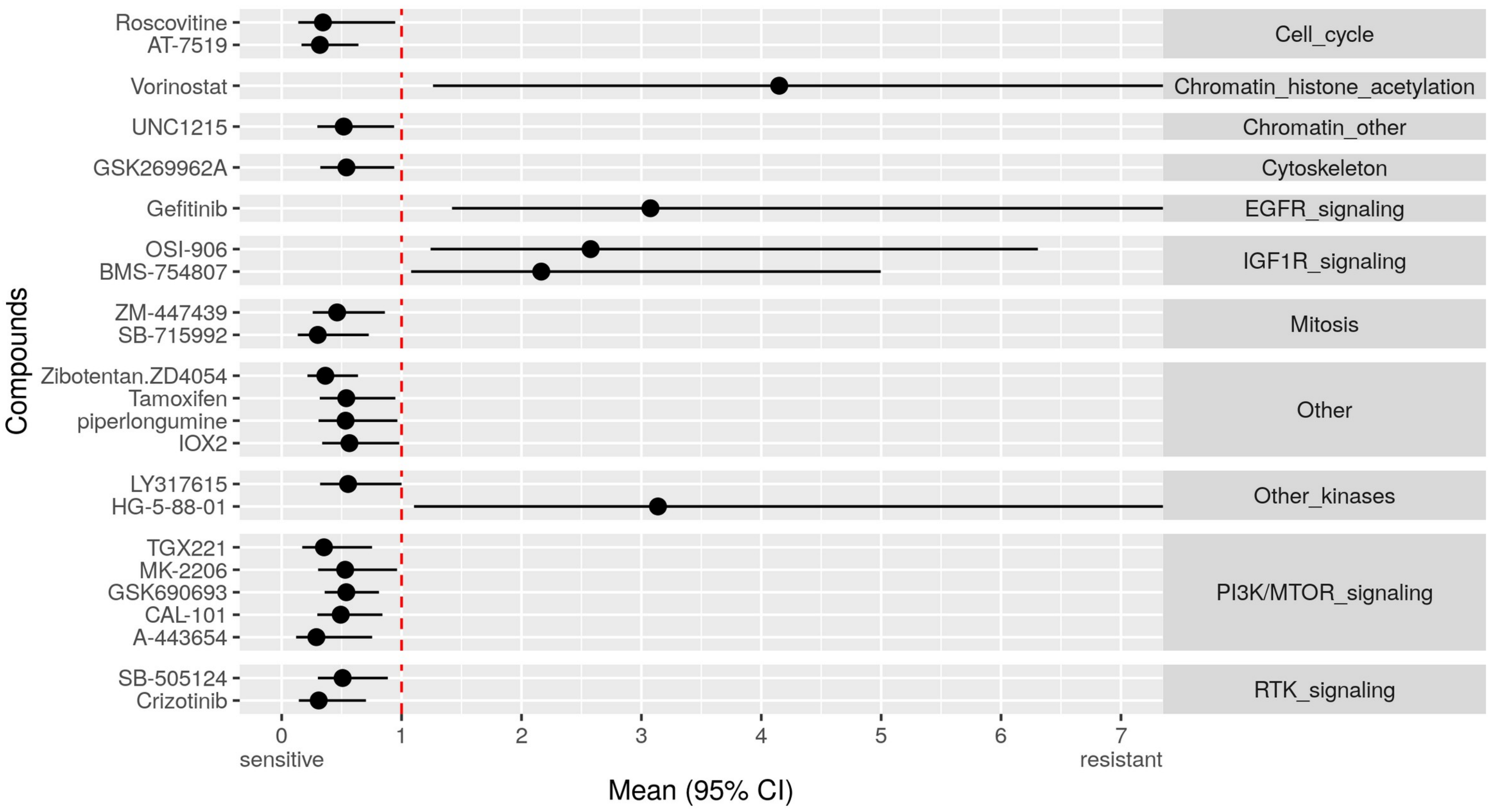

C

Top 10 most frequently mutated genes in Breast Invasive Carcinoma (TCGA, Firehose Legacy)

Mutated Genes (982 profiled samples)			
Gene	# Mut	#	Freq
PIK3CA	355	319	32.5%
TP53	304	301	30.7%
CDH1	114	112	11.4%
GATA3	101	97	9.9%
MAP3K1	98	71	7.2%
KMT2C	83	70	7.1%
NCOR1	42	40	4.1%
PTEN	37	35	3.6%
SPEN	44	33	3.4%
MAP2K4	32	32	3.3%

D

Breast Invasive Carcinoma (TCGA Firehose legacy)

A	B	Log2 Odds Ratio	p-Value	q-Value	Tendency
TP53	GATA3	1.604	<0.001	**<0.001**	Co-occurrence
NCOR1	MAP2K4	1.790	<0.001	**<0.001**	Co-occurrence
PIK3CA	GATA3	-1.416	<0.001	**<0.001**	Mutual exclusivity
TP53	PTEN	1.556	<0.001	**<0.001**	Co-occurrence
PIK3CA	PTEN	-1.516	<0.001	**0.001**	Mutual exclusivity
TP53	CDH1	-1.505	<0.001	**0.002**	Mutual exclusivity
GATA3	PTEN	1.331	<0.001	**0.002**	Co-occurrence
GATA3	KMT2C	0.926	0.006	**0.029**	Co-occurrence
KMT2C	NCOR1	1.020	0.006	**0.029**	Co-occurrence

Cancer Cell Line Encyclopedia (Broad, 2019)

A	B	Log2 Odds Ratio	p-Value	q-Value	Tendency
NCOR1	MAP2K4	>3	<0.001	**<0.001**	Co-occurrence
TP53	MAP2K4	2.882	<0.001	**<0.001**	Co-occurrence
TP53	NCOR1	1.641	<0.001	**<0.001**	Co-occurrence
GATA3	PTEN	1.213	<0.001	**<0.001**	Co-occurrence
GATA3	NCOR1	1.230	<0.001	**<0.001**	Co-occurrence
MAP3K1	NCOR1	1.260	<0.001	**<0.001**	Co-occurrence

E

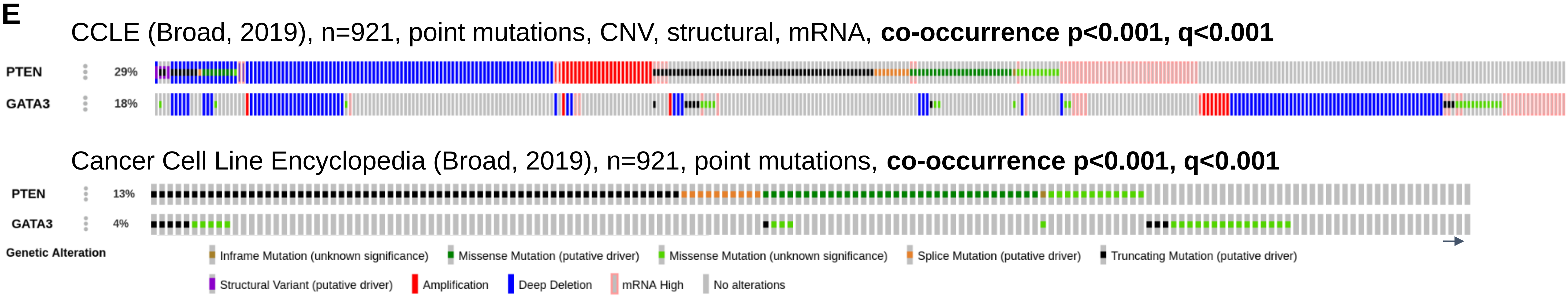

Supplementary Figure 6. *PTEN* and *GATA3*. **(A)** Drug sensitivity of the CCLE cell lines associated with deactivating changes (homozygous deletions, splice variants, frameshift and nonsense mutations) in *PTEN*. **(B)** Odds ratios (Fisher's test p<0.05) of significant single compounds. **(C)** Co-mutation of *PTEN* and *GATA3* in breast tumors and cancer cell lines. 10 most frequently mutated genes within Breast Invasive Carcinoma (TCGA, Firehose legacy; point mutations, www.cbioportal.org) and thereof **(D)** significant co-occurrence and mutual exclusivity (point mutations, structural and mRNA alterations) in breast cancer and the CCLE. **(E)** Co-occurrence of *PTEN* and *GATA3* genetic changes in CCLE significant also for point mutations only (excluding structural and mRNA changes to rule out that co-occurrence is based solely on the localization on the same chromosome arm). Arrow indicates the presence of more wild type samples not depicted.
