# Supplementary material for: Interactive webtool for analyzing drug sensitivity and resistance associated with genetic signatures of cancer cell lines
Source: J Cancer Res Clin Oncol. 2022 Dec 6;149(9):5539–45. doi: 10.1007/s00432-022-04503-2 (PMC10356876; doi:10.1007/s00432-022-04503-2)
Supplement: Supplementary file 2 — Supplementary file2 (DOCX 13 KB) [file 432_2022_4503_MOESM2_ESM.docx]

**Supplementary Tables**

**Supplementary Table 1.** Data sources.

| **Data** | **Source** |
| --- | --- |
| The GDSC drug response data set “cell-line/drug combinations classification (resistant/sensitive) form”, updated August 2020 | https://www.cancerrxgene.org/gdsc1000/GDSC1000_WebResources/Drug_screening_data.html |
| Detailed information file on drugs “Compounds-annotation” | https://www.cancerrxgene.org/downloads/bulk_download |
| Genetic alterations queried for the “Cancer Cell Line Encyclopedia (Broad, 2019)”, July 2020 to October 2021. | www.cbioportal.org |

**Supplementary Table 2. Sensitivity and resistance to PARPi in cell lines with ‘BRCAness’.** Genetically altered group defined by putatively deactivating changes (homozygous deletions, splice variants, frame shift, nonsense mutations) in at least one of the following genes: *BRCA1, BRCA2, ATM, ATR, RAD51C, RAD51D*. Compared to wild types across entities and breast cancer cell lines only (n=45). Full results: Results/BRCAness/across_entities/BRCAness_tables_across_entities.xlsx

| **Drug** | **cell line entity** | **Observed effect** | **OR** | **fisher_p** | **chi_p** | **lower** | **upper** |
| --- | --- | --- | --- | --- | --- | --- | --- |
| Olaparib | across entities | No significance | 1.08 | 0.80 | 0.74 | 0.66 | 1.85 |
|  | breast | No significance | 0.83 | 1 | 0.86 | 0.08 | 8.6 |
| AG-014699 (rucaparib) | across entities | No significance | 1.13 | 0.77 | 0.66 | 0.65 | 2.07 |
|  | breast | No significance |  | 0.49 |  |  |  |
| BMN-673 (talazoparib) | across entities | No significance | 1.14 | 0.71 | 0.59 | 0.70 | 1.92 |
|  | breast | No significance |  | 1 |  |  |  |
| ABT-888  (veliparib) | across entities | No significance | 1.28 | 0.43 | 0.36 | 0.76 | 2.26 |
|  | breast | No significance |  | 1 |  |  |  |

**Supplementary Table 3. Odds ratio for resistance to the Akt inhibitor GSK690693 for *PIK3CA* mutated cancer cell lines.** OR: odds ratio. Lower, upper: boundaries of 95% confidence intervals, wt: wildtype, mut: mutated/altered, s: sensitive, r: resistant.

| *PIK3CA* mutation | OR | Observed effect | fisher_p | chi_p | lower | upper | wt, s | wt, r | mut, s | mut, r |
| --- | --- | --- | --- | --- | --- | --- | --- | --- | --- | --- |
| C420, E542, H1047 | 0.28 | Sensitivity | 0.00019 | 8.46E-05 | 0.14 | 0.55 | 236 | 473 | 35 | 29 |
| H1047 | 0.21 | Sensitivity | 0.00028 | 0.00014 | 0.08 | 0.50 | 236 | 473 | 17 | 7 |
| E542K, E545K | 0.81 | No significance | 0.68 | 0.60 | 0.38 | 1.82 | 236 | 473 | 11 | 18 |

**Supplementary Table 4. Drug response to 21 compounds in the PI3K/MTOR signaling pathway for *PTEN* deactivated and *GATA3* co-mutated cell lines.** Sums of cell lines tested with various compounds of the pathway. *PTEN* deactivation: CNV down, mRNA low, frameshift, nonsense, splice (the latter three types are known to be loss of function). *GATA3* activation: CNV up, mRNA high. *GATA3* deactivation: CNV down, mRNA low. S: sensitive. R: resistant. Mut: mutated. Wt: wild type. OR: odds ratio for resistance in mutated cell lines. Lower, upper: boundaries of 95% confidence intervals.

| **Genes** | **S mut** | **R mut** | **S wt** | **R wt** | **Effect** | **OR** | **fisher_p** | **chi_p** | **lower** | **upper** |
| --- | --- | --- | --- | --- | --- | --- | --- | --- | --- | --- |
| ***PTEN* deactivation** | 305 | 1724 | 1483 | 11213 | Sensitivity | 0.75 | 2.9E-05 | 1.8E-05 | 0.66 | 0.86 |
| ***PTEN* deactivation and *GATA3* activation** | 47 | 514 | 1969 | 13545 | Resistance | 1.59 | 0.0018 | 0.0024 | 1.18 | 2.17 |
| ***PTEN* deactivation and *GATA3* deactivation** | 51 | 484 | 1969 | 13545 | No effect | 1.38 | 0.03 | 0.03 | 1.04 | 1.87 |

**Supplementary Table 5. Olaparib sensitivity and resistance in cell lines with alterations of *ABCB1*.** Activating: DNA amplification, mRNA high. Inactivating: DNA deletion, mRNA low, nonsense and frameshift mutations.

| ***ABCB1*** | **Sensitive** | **Resistant** | **Percent sensitive** |
| --- | --- | --- | --- |
| Activation | 0 | 76 | 0 |
| Unchanged | 88 | 622 | 12.39 |
| Inactivation | 3 | 18 | 14.29 |
